# Validation of ICD-9 Codes for Stable Miscarriage in the Emergency Department

**DOI:** 10.5811/westjem.2015.4.24946

**Published:** 2015-06-22

**Authors:** Kelly E. Quinley, Ailsa Falck, Michael J. Kallan, Elizabeth M. Datner, Brendan G. Carr, Courtney A. Schreiber

**Affiliations:** * Highland Hospital of Alameda Health System, Department of Emergency Medicine, Oakland, California; † James Madison University, Harrisonburg, Virginia; ‡ Perelman School of Medicine, University of Pennsylvania, Center for Clinical Epidemiology and Biostatistics, Philadelphia, Pennsylvania; § Perelman School of Medicine, University of Pennsylvania, Department of Emergency Medicine, Philadelphia, Pennsylvania; ¶ Thomas Jefferson University, Department of Emergency Medicine, Philadelphia, Pennsylvania; || Perelman School of Medicine, University of Pennsylvania, Department of Obstetrics and Gynecology, Philadelphia, Pennsylvania

## Abstract

**Introduction:**

International Classification of Disease, Ninth Revision (ICD-9) diagnosis codes have not been validated for identifying cases of missed abortion where a pregnancy is no longer viable but the cervical os remains closed. Our goal was to assess whether ICD-9 code “632” for missed abortion has high sensitivity and positive predictive value (PPV) in identifying patients in the emergency department (ED) with cases of stable early pregnancy failure (EPF).

**Methods:**

We studied females ages 13–50 years presenting to the ED of an urban academic medical center. We approached our analysis from two perspectives, evaluating both the sensitivity and PPV of ICD-9 code “632” in identifying patients with stable EPF. All patients with chief complaints “pregnant and bleeding” or “pregnant and cramping” over a 12-month period were identified. We randomly reviewed two months of patient visits and calculated the sensitivity of ICD-9 code “632” for true cases of stable miscarriage. To establish the PPV of ICD-9 code “632” for capturing missed abortions, we identified patients whose visits from the same time period were assigned ICD-9 code “632,” and identified those with actual cases of stable EPF.

**Results:**

We reviewed 310 patient records (17.6% of 1,762 sampled). Thirteen of 31 patient records assigned ICD-9 code for missed abortion correctly identified cases of stable EPF (sensitivity=41.9%), and 140 of the 142 patients without EPF were not assigned the ICD-9 code “632”(specificity=98.6%). Of the 52 eligible patients identified by ICD-9 code “632,” 39 cases met the criteria for stable EPF (PPV=75.0%).

**Conclusion:**

ICD-9 code “632” has low sensitivity for identifying stable EPF, but its high specificity and moderately high PPV are valuable for studying cases of stable EPF in epidemiologic studies using administrative data.

## INTRODUCTION

Early pregnancy failure (EPF), commonly known as miscarriage, is frequently diagnosed in the emergency department (ED).^1^ In the United States, approximately 500,000 visits for vaginal bleeding during early pregnancy constitute 1.6% of all ED encounters each year, and recent trends have seen increasing numbers of ED visits nationally for symptomatic early pregnancy.^2^ The use of national or population-based databases for studying EPF management is limited by the ability to identify patients presenting with different classifications of EPF, which ultimately can dictate the treatment options available to these patients.^3^–^4^ Specifically, women presenting with cases of stable EPF, also known as missed abortion, are candidates for multiple treatment options, including expectant management, medical management, or uterine evacuation either at the bedside in the ED or as an outpatient.^5^

To date, International Classification of Disease, Ninth Revision (ICD-9) diagnosis codes have not been validated for identifying cases of missed abortion, where a pregnancy is no longer viable, but the cervical os remains closed. These women tend to be more stable than those presenting with inevitable or incomplete abortion, where an open cervical os and active bleeding may prompt providers to prioritize urgent medical or surgical options to abate bleeding or infection risks. The ability to identify patients presenting with this specific subclass of EPF would allow for quality improvement and research initiatives targeting this common condition. Additionally, whether emergency providers and billing coders diligently or correctly label patients’ types of miscarriage (stable vs. inevitable vs. incomplete vs. complete abortion) has yet to be studied. Finally, the advent of the ICD-10 code classification system does not nullify the utility of future studies validating ICD-9 codes, as the medical field will undoubtedly continue to use large state- or government-sponsored databases of ICD-9 codes to retrospectively investigate clinical questions based on patient data coded during the ninth revision of the ICD system. Hence, ICD-9 codes continue to hold importance in the field of medical investigation. We therefore aimed to establish the ability of ICD-9 codes to identify patients with missed abortion in a single urban tertiary care hospital.

## METHODS

### Study Design and Data Sources

We completed a retrospective cohort study of patients who presented to the ED of a tertiary care urban academic center. Our goal was to determine the ability of diagnostic codes to identify patients presenting with stable non-viable pregnancy (i.e. missed abortion or stable early pregnancy failure) to the ED. We analyzed data from two perspectives in order to evaluate both the sensitivity and the positive predictive value (PPV) of ICD-9 code “632” for missed abortion in identifying stable miscarriages and retained products of conception following miscarriage.

We identified the population of interest from the hospital’s electronic medical record (EMR) based on chief complaint and ICD-9 codes from patient encounters between June 1, 2011 and May 31, 2012, and performed chart reviews of these encounters.

Prior research has illustrated that emergency medicine residents and attending physicians can accurately determine gestational age in first trimester pregnancy using bedside ultrasound studies.^6^ Estimated gestational age of pregnancy was determined by either an ultrasound evaluation conducted by an emergency medicine resident or attending physician at patient bedside, or by formal ultrasound completed by an ultrasound technologist with interpretation by a radiologist, where crown-rump length was correlated with gestational age based on averages of fetal size by weeks.

This study was approved by local institutional review.

### Nomenclature and Definitions

The term fetal demise refers to the process by which embryonic tissue begins to develop but then loses viability or dies.^7^ We defined missed abortion as pregnancy failure in patients with a previously identified pregnancy, a positive pregnancy test and a closed cervical os on bimanual exam, with the absence of fetal cardiac activity on ultrasound exam at six weeks or greater gestational age.^8^ This definition of missed abortion diverges slightly from the textbook definition of missed abortion, which originated prior to ultrasound diagnosis and was defined as fetal demise recognized eight weeks prior without passage of products of conception, and where the use of the word ‘missed’ referred to an abnormal pregnancy where the uterus had missed that the intrauterine contents needed to be expelled.^3^,^7^ In the absence of ultrasound, the only means of diagnosis was a discrepancy between uterine size and menstrual cycles.^7^ With the current use of ultrasonographic parameters in diagnosing pregnancy failure, the textbook definition with recognized pregnancy failure eight weeks earlier is not commonly fulfilled, and ED visits are nevertheless coded as missed abortion with ICD-9 codes following a single visit.^9^ Women with retained products of conception following a previously identified miscarriage were also included in our cohort.

### Inclusion and Exclusion Criteria

We studied female patients ages 13 to 50 years presenting to the ED with early pregnancy failure. We excluded patients whose ultrasound examinations identified a fetal pole with gestational age less than six weeks (due to the difficult nature of definitively diagnosing a failed pregnancy this early), as well as women with pregnancies over 22 weeks gestation. Women whose cervical os status was not included in their patient record were also excluded, as absence of this information precluded the determination of whether the patient’s miscarriage was imminent or in progress (open os), or stable (closed os). The presence of vaginal bleeding was not a determining factor for study eligibility.

### Analysis

We performed a retrospective validation of ICD-9 code “632” for cases of missed abortion. We first aimed to determine the sensitivity of the ICD-9 code “632” (code diagnosis includes missed abortion with fetal death before 22 weeks of completed gestation, and retained products of conception not following elective abortion) in correctly identifying patients with true cases of stable miscarriage or retained products of conception following miscarriage. We identified patients who presented to the ED with a chief complaint of “pregnant and bleeding” or “pregnant and cramping” from June 1, 2011 through May 31, 2012 and pulled their records from the EMR. Using a random number generator (randomization tool Research Randomizer 4.0 [Urbaniak, GC & Plous, 2013]), we randomly selected two months of patient visits for review. Once cases meeting the study definition of missed abortion were identified, we calculated sensitivity of ICD-9 code “632” for identifying true cases of stable EPF.

With the goal of determining the predictive value of using ICD-9 code “632” to accurately identify cases of stable EPF, we aimed to determine the PPV of ICD-9 code “632” in correctly identifying patients who presented with missed abortion or retained products of conception following miscarriage. We queried the EMR over the same 12 months to identify patients whose encounters were labeled with ICD-9 code “632.” For this portion of the analysis, the limited number of charts labeled with ICD-9 code “632” prompted us to review 12 months of patient encounters instead of the random two-month sample that was used to evaluate the ICD-9 code’s sensitivity and specificity. We reviewed these records to determine which patients had actual cases of stable EPF, as well as which pregnancy-related ICD-9 codes were used to code these patient visits. The PPV of ICD-9 code 632 for capturing cases of stable miscarriage or retained products was then calculated.

We considered our definition of missed abortion to be the standard against which the accuracy of the ICD-9 codes assigned to the patient encounters should be judged. Descriptive statistics were calculated. We used frequencies and proportions to summarize categorical variables and means, and interquartile ranges were used to summarize continuous variables. ICD-9 codes and true diagnoses were tabulated, and sensitivities, specificities and PPVs were calculated. We made all tabulations and calculations using Stata 12.0 (Stata Corporation, College Station, TX).

A single reviewer who uses the EMR in regular clinical practice trained a second reviewer to use the EMR and conduct chart reviews. Both reviewers evaluated patient charts and used a single template to record ICD-9 codes and ultrasound results with documented viability of pregnancy and cervical os status, and to categorize patients as either having a stable miscarriage or another type of pregnancy failure.

## RESULTS

### Sensitivity and Specificity Findings

A total of 1,762 women with chief complaints of “pregnant and bleeding” or “pregnant and cramping” presented to the ED over the 12-month period. Of these, 310 patient records (a random sample of 17.6%) were reviewed, and 173 patients met study criteria for stable early pregnancy failure. The majority of subjects were black, with an average age of 26 years (Table 1). Just under thirty percent of these patients had previously experienced at least one miscarriage. No patients were found to have multiple gestations, and 31.2% of patients had visited an ED for a pregnancy-related complaint within one month of presentation to our academic medical center.

Of the 173 patient records meeting study inclusion criteria, 31 charts were assigned the ICD-9 code “632” for missed abortion, and medical chart review revealed 13 of these patients to have actual cases of stable EPF (sensitivity=41.9%). Of the 142 patients who did not have stable miscarriages, 140 patients were assigned ICD-9 codes other than code “632” (specificity=98.6%). These findings are illustrated in Table 2.

The majority of patient charts were labeled with ICD-9 codes for unspecified hemorrhage in early pregnancy (38.9%) or threatened abortion (27.8%). Table 3 illustrates the ICD-9 diagnosis codes other than “632” that were assigned to patients who had actual cases of missed abortion.

### Positive Predictive Value Findings

Of 1,762 women with chief complaints of “pregnant and bleeding” or “pregnant and cramping” presenting to the ED over the 12-month period, we identified 62 patient visits with ICD-9 code “632” for missed abortion. Fifty-two of these subjects were eligible for inclusion in the study, with nine excluded for lack of cervical os status mentioned in their record, and one whose pregnancy gestation was too young to determine viability via ultrasound and was therefore excluded. The majority of subjects were black, with an average age of 28 years (Table 4). Just under thirty percent of these patients had previously experienced at least one EPF, and 42.3% of patients had been seen at an ED for a pregnancy-related complaint within one month of presentation to our hospital.

Of the 52 study subjects whose visit was assigned ICD-9 code “632,” 39 patients had stable miscarriages (PPV=75.0%), as illustrated in Table 5. Most patients whose charts were incorrectly labeled with ICD-9 code “632” actually had cases of incomplete abortion (53.9%), where an open cervical os indicates an active expulsion of products of conception (Table 6).

## DISCUSSION

In this study we examined the ability of ICD-9 diagnostic codes to correctly identify patients with stable EPF. For patients presenting to an ED with symptomatic pregnancy, the ICD-9 code “623” for missed abortion, when defined as vaginal bleeding or abdominal cramping in the setting of a previously recognized pregnancy, is only 41.9% sensitive for accurately identifying cases of stable EPF. Conducting research on missed abortions with administrative data where ICD-9 code “632” is used to identify patients therefore has the potential to grossly underestimate the true number of patients seen in EDs with this diagnosis. However, a specificity of 98.6% and a PPV of 75.0% indicate that researchers can identify true cases of stable miscarriage by ICD-9 code “632” with a low rate of false positives. As such, the varying treatments, responses to treatment and complications of stable EPF can be studied in large epidemiologic studies when identifying subjects by diagnostic codes.

To the best of our knowledge, there have been no validations of miscarriage-related ICD-9 codes within the field of emergency medicine. This study is important for future epidemiologic research as well as quality-improvement initiatives for EPF treated in the ED setting. The relevance of these initiatives increase as more hospitals adopt the practice of having obstetrician gynecologist consultants treat miscarriage with uterine aspiration at the patient bedside in the ED. Furthermore, numerous ICD-9 codes exist for varying types of EPF (Table 7), and the presenting symptoms, level of critical acuity, management options, and associated costs are varied.

The 41.9% sensitivity we found for ICD-9 code “632” in accurately identifying cases of stable EPF begs the question as to why the sensitivity was found to be so low. Table 3 illustrates that most cases where missed abortions were classified under ICD-9 codes other than “632” were coded with either “640.93” (unspecified hemorrhage in early pregnancy, antepartum condition or complication) or “640.03” (threatened abortion). One commonality among these diagnosis codes is that they do not require the care provider to commit to deeming a pregnancy non-viable. This is perhaps owing to either a knowledge gap that ED providers may have in parameters for diagnosing EPF, especially following a single ultrasound study, or potentially an avoidance of making an EPF diagnosis owing to a perceived medical-legal risk of deeming a pregnancy failed if a provider is unsure of his or her abilities to do so. A third possibility is that emergency providers may leave the diagnosis of EPF to their obstetrician gynecologist consultants and may therefore document in a way that is non-committal with regards to the viability of the pregnancy. As this study was not designed to answer these questions, we can only postulate that these factors may have played a role in our findings, but more studies are needed to explore these possibilities.

## LIMITATIONS

Our study has multiple limitations. We reviewed charts of patients whose pregnancies had been identified prior to presentation; therefore, our findings may not be representative of populations of women whose miscarriages are diagnosed in the ED. All ICD-9 codes in this study were assigned by medical coders after the patient encounters had come to a close. Though this limits the generalizability of the study findings to hospitals that do not use coders and instead have care providers select or determine the ICD-9 codes themselves, we assumed that there would likely be less variability between people who assign ICD-9 codes as a profession than between residents and attending providers. Our subjects were collected from a single institution, which may limit the generalizability of our findings to other hospitals, though our data may still be useful when considering other academic tertiary care EDs where medical coders assign ICD codes. For our computation of the sensitivity and specificity of ICD-9 code “632,” we selected two months of patient visits out of a 12-month period, so it is possible that secular trends may have biased results. Additionally, by identifying patients for inclusion in the study on the basis of presenting with a chief complaint of either “pregnant and bleeding” or “pregnant and cramping,” we likely missed a small number of cases of missed abortion in patients who presented to the ED without either of those chief complaints, which may have increased the sensitivity of our results. Including more symptoms associated with miscarriage, such as back pain or abdominal pain, could have afforded a more compete sample of miscarrying patients. This study illustrates the importance for emergency providers of performing and documenting an accurate cervical exam when appropriate, as cervical os status has implications for the management options available for different classifications of pregnancy failure. Finally, the inter-rater reliability of chart reviewers was not evaluated, which had the potential to bias results if one reviewer had stricter standards for determining a stable miscarriage. However, the definition of a missed abortion was objective and equal for both reviewers, which we believe tempered any subjectivity in determining cases of missed abortion.

## CONCLUSION

The ICD-9 code “632” for missed abortion has a low sensitivity for identifying women with stable EPF, which limits its use in benchmarking the number of cases when using administrative databases. Another approach to identifying missed abortion using ICD-9 codes is therefore needed. However, the high specificity and moderately high PPV of ICD-9 code “632” for identifying women presenting to the ED with missed abortion illustrate its utility in identifying true cases of stable miscarriage, so that subsequent interventions and complications can be examined in future epidemiologic studies.

## Figures and Tables

**Table 1:** General demographics of patients presenting with chief complaint of “pregnant and bleeding” for sensitivity and specificity analysis (n=173).

Variable	n (% or range)
Age, mean, years	26 (14–43)
Race	
Black	146 (84.4%)
Caucasian	8 (4.6%)
Latina	4 (2.3%)
Asian	11 (6.4%)
Arab	4 (2.3%)
Gravida, mean (±SD)	3.4 (±2.2)
Patients with at least one previous miscarriage	
Yes	51 (29.5%)
No	122 (70.5%)
Presence of vaginal bleeding*	
Yes	139 (80.3%)
No	33 (19.1%)
Unknown	1 (0.6%)
Estimated gestational age, mean (range)	9 weeks 2 days (21–122d)
Patients with emergency department visit to any hospital for pregnancy-related complaint within 1 month of presentation	
Yes	54 (31.2%)
No	119 (68.8%)

* Not all charts had presence of vaginal bleeding listed.

**Table 2:** Sensitivity and specificity findings.

	Patient presentation met definition of ICD-9 “632” code	
	
Patient encounter assigned ICD-9 code “632”	Yes	No
Yes	13	2
No	18	140
	Sensitivity=41.9%	Specificity=98.6%

*ICD-9,* International Classification of Disease, Ninth Revision

* Non-viable pregnancy or retained products of conception with closed cervix.

**Table 3:** Pregnancy-related ICD-9 codes for patients with missed abortion but whose chart was labeled with ICD-9 code other than “632” (n=18).

ICD-9 code	ICD-9 diagnosis	n (%) of missed abortion with ICD-9 code other than “632”
640.93	Unspecified hemorrhage in early pregnancy, antepartum condition or complication	7 (38.9%)
640.03	Threatened abortion	5 (27.8%)
631	Other abnormal product of conception	1 (5.6%)
633	Ectopic pregnancy	1 (5.6%)
634.9	Spontaneous abortion without mention of complications – unspecified stage	1 (5.6%)
640.83	Other specified hemorrhage in early pregnancy	1 (5.6%)
641.93	Unspecified antepartum hemorrhage, antepartum condition or complication	1 (5.6%)
649.53	Spotting pregnancy, antepartum condition or complication	1 (5.6%)

*ICD-9,* International Classification of Disease, Ninth Revision

**Table 4:** General patient demographics for positive predictive value analysis (n=52).

Variable	n (% or range)
Age, mean, years	28 (17–44)
Race	
Black	41 (78.9%)
Caucasian	4 (7.7%)
Latina	2 (3.9%)
Asian	5 (9.6%)
Gravida, mean (±SD)	3.6 (±2.8)
Patients with at least one previous miscarriage	
Yes	12 (28.6%)
No	30 (71.4%)
Presence of vaginal bleeding^*^	
Yes	42 (80.8%)
No	9 (17.3%)
Unknown	1 (1.9%)
Estimated gestational age, mean (range)	9 weeks 4 days (39–122d)
Patients with emergency department visit to any hospital for pregnancy-related complaint within 1 month of presentation	
Yes	22 (42.3%)
No	29 (55.8%)
Unknown	1 (1.9%)

**Table 5:** Positive predictive value analysis.

	Patient presentation met definition of ICD-9 “632” code		
	
Patient encounter assigned ICD-9 code “632”	Yes	No	
Yes	39	13	PPV=75.0%
No	0	0	NPV=not applicable

*ICD-9,* International Classification of Disease, Ninth Revision; *PPV*, positive predictive value; *NPV*, negative predictive value

* Non-viable pregnancy or retained products of conception with closed cervix.

**Table 6:** Pregnancy-related diagnoses and corresponding ICD-9 codes for patients without missed abortion but whose chart was labeled with ICD-9 code “632” (n=13).

ICD-9 Code	ICD-9 diagnosis	n (%)
634.91	Spontaneous abortion without mention of complications - incomplete	7 (53.9%)
634.92	Spontaneous abortion without mention of complications - complete	2 (15.4%)
633	Ectopic pregnancy	1 (7.7%)
637	Unspecified abortion, retained products of conception following abortion	1 (7.7%)
637.9	Abortion (complete, incomplete, inevitable or with retained products of conception)	1 (7.7%)
639	Genital tract and pelvic infection, including endometritis	1 (7.7%)

*ICD-9,* International Classification of Disease, Ninth Revision

**Table 7:** Miscarriage-related ICD-9 codes.

ICD-9 code	ICD-9 diagnosis
631	Other abnormal product of conception
632	Missed abortion (early fetal death before 22 completed weeks of gestation), retained products of conception, not following spontaneous or induced abortion or delivery
634.9	Spontaneous abortion without mention of complications - unspecified stage
634.91	Spontaneous abortion without mention of complications - incomplete
634.92	Spontaneous abortion without mention of complications - complete
637	Unspecified abortion, retained products of conception following abortion
637.9	Abortion (complete, incomplete, inevitable with or without retained products of conception)
637.91	Unspecified abortion, without mention of complication - incomplete
637.92	Unspecified abortion, without mention of complication - complete
640	Threatened abortion
640.8	Other specified hemorrhage in early pregnancy
640.9	Unspecified hemorrhage in early pregnancy
641.93	Unspecified antepartum hemorrhage
646.83	Other specified complications of pregnancy
649.53	Spotting complicating pregnancy, antepartum condition or complication

*ICD-9,* International Classification of Disease, Ninth Revision
